# Silent Burden of Urinary Tract Infections in Intermittent Catheter Users with Neurological Disorders: A Scoping Review

**DOI:** 10.3390/diseases14020058

**Published:** 2026-02-03

**Authors:** Floriana D’Ambrosio, Ciro Pappalardo, Anna Scardigno, Manuel Del Medico, Pietro Eric Risuleo, Francesca Orsini, Roberto Ricciardi, Elisabetta De Vito, Walter Ricciardi, Giovanna Elisa Calabrò

**Affiliations:** 1 Department of Life Sciences, Health and Health Professions, Link Campus University, 00165 Rome, Italy; floriana.dambrosio@unilink.it; 2 Section of Hygiene, Department of Life Sciences and Public Health, Università Cattolica del Sacro Cuore, 00168 Rome, Italy; cirpap16@gmail.com (C.P.); manuel.delmedico01@icatt.it (M.D.M.); pietroeric.risuleo01@icatt.it (P.E.R.); walter.ricciardi@unicatt.it (W.R.); 3 Laboratory of Pharmacoepidemiology and Human Nutrition, Department of Health Policy, Istituto di Ricerche Farmacologiche Mario Negri IRCCS, 20156 Milan, Italy; francesca.orsini@marionegri.it; 4 VIHTALI (Value in Health Technology and Academy for Leadership & Innovation), Spin-Off of Università Cattolica del Sacro Cuore, 00168 Rome, Italy; robertoricciardi.mail@gmail.com; 5 Department of Human Sciences, Society and Health, University of Cassino and Southern Lazio, 03043 Cassino, Italy; elisabetta.devito@unicas.it; 6 European University of Technology EUt+, European Union, 03043 Cassino, Italy

**Keywords:** epidemiology, scoping review, public health, urinary tract infections, intermittent catheterization, spinal cord injury, multiple sclerosis, neurogenic bladder

## Abstract

**Objective:** To map and synthesize the published literature on the epidemiological burden of urinary tract infections (UTIs) in adults with spinal cord injury (SCI) or multiple sclerosis (MS) using intermittent catheterization (IC). **Methods:** We conducted a comprehensive literature review following PRISMA guidelines, searching PubMed, Scopus, and Web of Science for studies published since 2014. A total of 30 studies met the inclusion criteria. **Results:** Reported UTI incidence varied widely from 24% to 93.1%, highlighting significant heterogeneity across the evidence base. Annually, 15–17% of patients experienced 4–6 UTIs, and up to 16.4% required hospitalization for UTI-related complications. A critical evidence gap was exposed, with only one study focusing specifically on the MS population. **Conclusions**: Despite its clinical benefits, IC remains underutilized and inconsistently supported. Addressing systemic delivery gaps is essential. UTIs in neurogenic bladder care should be recognized as a modifiable public health issue requiring equity-driven interventions and strengthened implementation frameworks. This review underscores the urgent need for methodologically rigorous research to establish clear best practices.

## 1. Introduction

Neurogenic lower urinary tract dysfunction (NLUTD) affects most individuals with spinal cord injury (SCI) and a significant proportion of those with multiple sclerosis (MS), often resulting in urinary tract infections (UTIs), incontinence, and renal complications [1]. The prevalence of NLUTD is particularly pronounced in the SCI population, where it affects up to 80% of individuals, while approximately 25% of patients with MS develop neurogenic bladder dysfunction over the course of their illness [2,3]. Intermittent catheterization (IC), especially clean IC (CIC), is widely endorsed for managing NLUTD, with benefits including improved autonomy and reduced long-term morbidity [4].

Despite this, UTIs remain highly prevalent among IC users, with reported incidence reaching 36% to 60% in some cohorts [5,6]. These infections reduce quality of life, increase hospitalizations, and generate substantial healthcare costs—particularly during the first year after bladder dysfunction [7].

Risk factors such as catheter reuse, poor hygiene, and limited access to single-use hydrophilic catheters are well documented [8], while structured education, long-term follow-up, and antimicrobial resistance surveillance remain underutilized [8,9].

Furthermore, challenges persist with adherence to the practice, with studies reporting dropout rates as high as 50% within the first five years after initiation and with factors such as age, gender, manual dexterity, and psychological barriers playing a significant role [10,11].

By examining the current evidence and identifying gaps in knowledge, this review aims to map and synthesize the existing literature on the epidemiological burden of UTIs among MS and SCI patients using IC. We seek to highlight the scale of the problem, identify common risk factors reported in the literature, and expose critical evidence gaps to guide future high-quality research. The ultimate goal is to inform clinical directions and public health strategies aimed at reducing infection rates and improving patient outcomes.

## 2. Materials and Methods

A comprehensive literature search was conducted. The reporting of this review was structured according to the “PRISMA Extension for Scoping Reviews (PRISMA-ScR)” guidelines (Table S1) [12]. However, the review and the protocol were not registered.

### 2.1. Search Strategy

The process of the literature search involved consulting three scientific databases: PubMed, Web of Science (WoS), Scopus. The following search string was used on PubMed: (Intermittent AND (catheterisation OR catheterization) AND (“urinary tract infection” OR “urinary tract infections” OR UTI OR UTIs)) OR (CAUTI OR CA-UTI OR “catheter-associated UTI” OR “Catheter-associated urinary tract infection” OR “Catheter-associated urinary tract infections”). This search string was adapted for WoS and Scopus. Searches were limited to the English language, published in the last ten years, involving adults (≥18 years) with MS or SCI.

The research question was defined according to the PICO (Population, Intervention, Comparator, Outcomes) framework (Table 1): “In adults with SCI or MS who use intermittent catheterization, what is the reported epidemiological burden of UTIs, and what risk factors and catheterization practices are associated with UTIs occurrence?”

Inclusion criteria for the screened studies were the following: (1) study population included adults aged ≥18 years affected by MS or SCI, (2) primary studies and systematic reviews, (3) articles written in English, and (4) articles published since 2014. We excluded non-English publications, studies other than primary research or systematic reviews, inaccessible full texts, studies lacking relevant data, and those unrelated to our PICO.

### 2.2. Data Extraction and Data Analysis

The search was conducted on 17 March 2025. Retrieved records were compiled in Excel, duplicates were removed, and screened in two stages, first by title/abstract, then by full text, according to predefined eligibility criteria.

No articles were added to our review as a result of snowballing.

Six researchers (F.D’A., M.D.M., F.O., C.P., P.E.R, A.S.) independently screened all results and excluded irrelevant articles screening by titles and abstracts.

Full texts were obtained and independently assessed by the reviewers. Publications were excluded only if all the reviewers agreed, and any discrepancies were resolved through discussion or senior researcher (G.E.C.) involvement.

Three researchers (C.P., M.D.M., P.E.R.) extracted data from all included publications using a standardized Excel spreadsheet. Extracted data were organized into an epidemiological and a risk factor table, including the following study details: author, year, country, type, sample/population, pathology, IC type/characteristics, UTI percentage, risk factors, and key findings.

Data were extracted as reported in the original studies; no conversions or imputations were performed. When multiple measures of UTI were reported (e.g., symptomatic, febrile), all were extracted and clearly labeled.

## 3. Results

### 3.1. Study Selection

**Figure 1:**
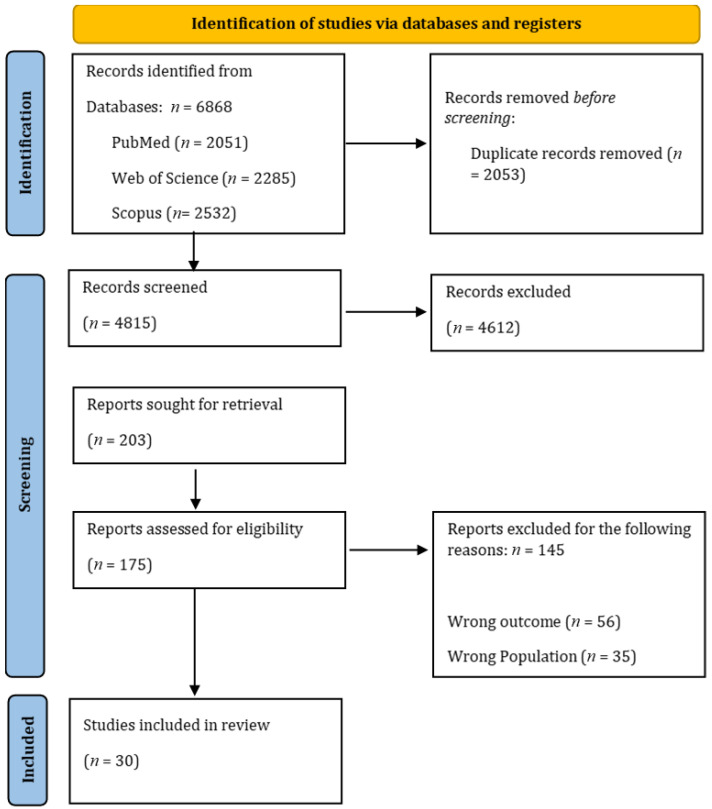
PRISMA flow diagram of included studies.

The search identified 6868 records. After the screening phases, 30 articles were ultimately included. The study selection process is illustrated in detail in Figure 1.

### 3.2. Study Characteristics

The included articles consisted of 29 primary studies (96.7%) (Table 2) and one systematic review (3.3%) [13]. Primary studies included eight cross-sectional studies (26.4%) [8,11,14,15,16,17,18,19], eight prospective (26.4%) [20,21,22,23,24,25,26,27], and twelve retrospective (39.6%) [3,28,29,30,31,32,33,34,35,36,37,38], along with one mixed-method study (3.3%) [10].

The highest proportion of studies were conducted in the USA (six, 20%) [11,13,21,22,27,30], followed by China (four, 13.2%) [18,29,33,36], Canada [17,20,39] and Taiwan [15,37,38] (three each, 10%), Turkey [3,14] and Japan [19,28] (two each, 6.6%). Other countries included Australia [24], Germany, The Netherlands [31], India [25], Serbia [34], Saudi Arabia [35], South Korea [32], Sweden [26], Switzerland [23], Tanzania [16], and UK [10] (3.3% each).

The sample sizes ranged from 31 [25] to 2924 [18] participants. Publication years included three studies published in 2014 (10%) [14,15,18], two in 2015 (6.6%) [30,39], one in 2016 (3.3%) [3], one in 2017 (3.3%) [20], one in 2018 (3.3%) [13], six in 2019 (20%) [10,21,22,23,24,33], six in 2020 (20%) [11,16,25,26,27,40], two in 2021 (6.6%) [17,32], two in 2022 (6.6%) [31,38], three in 2023 (10%) [18,19,29], and four in 2024 (13.2%) [34,35,36,37].

The majority of the included studies focused only on patients with SCI, comprising 29 studies (96.7%) [3,11,13,14,15,16,17,18,19,20,21,22,23,24,25,26,27,28,29,30,31,32,33,34,35,36,37,38,39], while MS patients were examined only in one study (3.3%) [10].

### 3.3. Epidemiological Burden of UTIs

The epidemiological burden of UTIs was assessed using varied methodologies and outcome measures, reflecting heterogeneity in populations, clinical practices, and case definitions.

#### 3.3.1. SCI Patients

Among the 29/30 (96.7%) studies focusing on SCI patients, UTI incidence was the most frequently reported outcome [13,15,16,17,18,19,20,25,29,31,33,34,35,37], with rates ranging from 24% [29] to 93.1% [18].

Five studies (16.6%) [11,21,22,26,27] assessed the annual number of UTIs. These revealed that 36.0% [26] to 49.0% [27] experienced 1–3 UTIs per year, 15% [21] to 16.7% [27] experienced 4–6 UTIs annually, and 10.2% [21] to 12.4% [27] more than six UTIs per year. Two studies found that 27.8% [22] and 34.0% [11] of patients experienced four or more UTIs annually.

Liu et al. [18] found that 20.4% of patients using Clean Intermittent Catheterization (CIC) < 1/day experienced UTIs, while the incidence rose to 74.5% for those using CIC 1–6/day, and up to 93.1% for those using CIC > 6 times per day. Additionally, these authors found that patients who reported difficulty inserting the catheter into the urethra had more than twice the odds of developing a UTI (OR = 2.30; 95% CI: 1.30–4.06). Likewise, individuals experiencing urinary incontinence at least once daily (OR = 2.17; 95% CI: 1.15–4.08) and those who had switched from CIC to another approach (OR = 2.36; 95% CI: 1.18–4.71) were at higher risk.

Berger et al. [31] reported an incidence rate of 31.46 UTIs per 100 person-months (PMs) among IC users, while Xing et al. [29] reported a rate of 1.31 events per 100 person-days, considering both sterile and clean catheterization techniques. Moreover, Hennessey et al. [24], assessed UTI incidence per 1000 days, indicating 6.84 cases in intermittent self-catheterization (ISC) users, while Cheng et al. [37] reported a monthly incidence of 0.03 UTIs/month.

Five studies (16.6%) [3,16,25,28,35] adopted differential outcomes. Neyaz et al. [25] described an incidence of symptomatic UTIs (sUTI) of 2.29 episodes per patient per year. Yilmaz et al. [3] observed 36.7% sUTI prevalence, while Ali et al. [35] reported sUTI rates of 46.6% for hydrophilic-coated and 79.4% for uncoated catheter users. Mukai et al. [28] described that 25.8% of CIC users experienced febrile episodes associated to UTI (fUTIs) and showed in further analyses that male gender (HR = 3.08; 95% CI: 1.04–9.15; *p* = 0.0431) and an ASIA impairment scale (a classification of SCI severity) [41] of C or worse (indicating moderate to severe impairment) (HR = 0.997; 95% CI: 0.994–1.000; *p* = 0.0266) were significantly associated with fUTI occurrence. Nade et al. [16] reported fUTIs in 39.2% of patients performing both self and non-self-catheterization.

Recurrent UTIs (RUTIs), defined as ≥2 UTIs within six months or ≥3 UTIs within one year, were reported in 2/30 (6.6%) studies. Liu et al. [18] attested a prevalence of 53.5%, while Chen et al. [38] identified RUTIs in 70.4% of CIC users.

Sekido et al. [19] assessed multiple outcomes, reporting an average of 2.8 symptomatic UTIs (sUTIs) per patient per year (SD 4.95), affecting 52.2% of patients. fUTIs were documented in 33.2% of patients, averaging 9.0 episodes annually (SD 2.14). Furthermore, standardized healthcare provider-diagnosed symptomatic UTIs (hcp-sUTIs) occurred at a mean rate of 0.89 episodes per patient-year (SD 2.83). Recurrent UTIs (RUTIs) were identified in 34.0% of ISC users, with 10.9% experiencing recurrent febrile UTIs and 20.6% recurrent hcp-sUTIs. Comparing reusable versus single-use catheters, the authors found sUTIs in 55.1% vs. 48.6%, fUTIs in 33.8% vs. 32.4%, and hcp-sUTIs in 24.8% vs. 23.4%, respectively.

#### 3.3.2. Impact of Catheter Type, Hygiene, and Education

Eight studies (26.7%) [3,19,23,32,34,35,36,39] evaluated the effects of catheter types, hygiene, and education.

UTI frequencies ranged from 22.2% [32] to 62.7% [34] for Intermittent Self Catheterization (ISC) and from 20.3% [32] to 60.0% [23] for assisted IC. Anderson et al. [23] reported higher incidence rate ratios (IRR) for assisted IC (6.05, 95% CI 2.63–13.94) vs. ISC (5.16, 95% CI 2.31–11.52).

Krassioukov et al. [39] found that single-use catheters were significantly associated with fewer UTIs per year (1 ± 1) compared to reusable catheters (4 ± 3), suggesting that the latter may represent a risk factor for UTI. On the other hand, Sekido et al. [19] reported no statistically significant differences in sUTI incidence or frequency between reusable catheters (55.1%) and single-use catheters (48.6%).

Yildiz et al. [14] instead, evaluated the aseptic technique, finding a 21.9% UTI incidence.

Ali et al. [35] conducted a retrospective cohort study on hydrophilic-coated catheter (HCC) and PVC catheter users with a symptomatic UTI incidence of 46.6% and 79.4%.

Kim et al. [32] examined UTI risk in relation to bladder management strategies. Compared to those who voided spontaneously, patients with urinary indwelling catheters (OR = 2.51; 95% CI: 1.43–4.41) and suprapubic catheters (OR = 4.42; 95% CI: 1.73–11.30) were at notably higher risk of developing UTIs.

Milicevic et al. [34] reported the highest UTI incidence among patients using ISC (62.7%), followed by those using assisted IC (17.2%), indwelling catheters (7.7%), reflex voiding (6.5%), and spontaneous voiding (6.0%).

Ultimately, Luo et al. [36] conducted a study evaluating the impact of structured health education for family members of patients using IC. Patients whose caregivers participated in the nurse-led educational program showed significantly lower incidence of UTIs (25%) compared to those who received standard care without family education (50%).

#### 3.3.3. Multiple Sclerosis Patients

Only one study [10] investigated UTIs in Clean Intermittent Catheterization (CIC) users affected by MS, focusing on the variables affecting the continuation or discontinuation of CIC use. This study involved 56 patients, with 43 identified as continuers and 13 as discontinuers at the 1-year follow-up. At baseline, 23% of discontinuers and 51% of continuers reported UTIs. At 8 months, 46% of both groups reported no UTIs, while 54% in each group experienced increased UTI frequency.

### 3.4. Risk Factors

We categorized the potential risk factors identified across the included studies using a framework adapted from Kennelly et al.’s UTI Risk Factor Model [8] (Table 3). This model groups factors into four domains: (1) general conditions, (2) intermittent catheterization, (3) user compliance and adherence, and (4) local urinary tract conditions. Table 3 summarizes the extracted risk factors according to these domains [8].

## 4. Discussion

### 4.1. Main Findings of This Study

This review reveals a critical and largely underrecognized burden: UTIs remain among the most prevalent complications in individuals with NLUTD, particularly those with SCI and MS. Across 30 studies, UTI incidence among IC users ranged from 24% [29] to 93.1% [18]. Interpreting these figures requires caution, as many studies failed to clearly distinguish between asymptomatic bacteriuria, which is common and often benign, and symptomatic or febrile UTIs. Despite this variability, the data indicate that approximately 15% of patients on average experienced 4–6 UTIs annually [25,26], and around 10% reported more than six [26,27]. Furthermore, up to 16.4% of patients experienced UTI-related hospitalizations [11].

The wide geographical distribution of the included studies emphasizes that this is not a localized phenomenon, but a systemic issue affecting multiple health systems. Although this problem disproportionately affects low-resource settings, inconsistencies in education, access, and support were also evident across higher-resource countries [20,36,42].

Despite its clinical advantages [43,44], IC is often hindered in its widespread adoption by high discontinuation rates and systemic barriers [45]. Factors such as limited insurance coverage, supply shortages within health systems, and financial constraints—particularly in low-resource settings—restrict access to single-use catheters and contribute to poor adherence, reducing the long-term effectiveness of IC [46,47]. Patient outcomes are also shaped by a range of external factors, including lack of training, inconsistent follow-up, and suboptimal patient engagement. This review identified some potential factors playing a role—most notably caregiver education, catheter reuse, and limited patient empowerment [18,28,32,36,39]. While promising evidence exists on the benefits of IC, further high-quality studies are needed to support more tailored, patient-centered applications that may improve UTI prevention. Currently, the evidence base is strongest for determining UTI incidence in male SCI patients within rehabilitation settings; in contrast, further research is needed for MS patients and community-dwelling individuals, where findings are more context-specific and variable.

While no single catheter technology emerged as definitively superior across all contexts, some studies suggest potential advantages of specific models in particular clinical scenarios [35]—warranting further investigation into context-sensitive device use.

### 4.2. What Is Already Known on This Topic

Current literature consistently confirms that UTIs are one of the most burdensome complications in individuals with NLUTD, associated with recurrent hospitalizations, diminished quality of life, and long-term health deterioration [1,48]. Global estimates for UTI prevalence in SCI populations range widely, from 10% to 68% [49], aligning with the burden observed in our review. The high prevalence of UTIs is also deeply rooted in the underlying pathophysiology of NLUTD. The AUA/SUFU and EUA guidelines highlight that regular bladder monitoring is essential, given that high intravesical pressure, poor compliance, and improper catheterization frequency are key determinants of infection risk [43,44].

Furthermore, there is currently a lack of consensus on UTI definitions. Literature highlights how distinguishing asymptomatic bacteriuria (ASB) from true infection remains challenging, particularly when impaired bladder sensation limits symptom reporting [8]. These challenges lead to multiple non-comparable operational definitions used in the published literature, contributing to the wide prevalence range observed across the literature and in our review. CIC is widely endorsed in clinical guidelines as a preferred strategy for bladder management over indwelling catheterization [43,44]. Its primary advantages include the preservation of renal function, a reduction in long-term complications such as upper urinary tract deterioration, and enhanced patient autonomy [50]. In addition, its cost-effectiveness has been demonstrated, with reductions in expenditures attributable to fewer complications and improved self-management [51]. However, these benefits must be weighed against potential disadvantages, such as the requirement for sufficient manual dexterity, the psychological burden of adherence, and the potential for urethral trauma, which can hinder long-term compliance [10,11].

IC implementation remains, therefore, unstandardized across care settings [52]. As Prieto et al. stated in 2021, it remains uncertain whether UTI rates are further affected by specific catheterization techniques (e.g., sterile vs. clean), catheter types (e.g., coated vs. uncoated), or even length—gaps that complicate clinical decision-making and highlight the need for more research [53].

Our findings do not challenge IC as the preferred bladder management strategy but underscore that its protective potential against UTIs is heavily context-dependent, requiring standardized protocols, education, and equitable access to optimize outcomes.

### 4.3. What This Study Adds

This study aimed to synthesize the broader epidemiological landscape of IC-related UTIs to reveal systemic failures in care delivery. While the existing literature often frames UTIs as a procedural complication, our synthesis identifies them as a “silent” public health crisis driven by structural barriers. By mapping clinical and non-clinical determinants alongside infection rates, this review provides a novel framework that reframes UTI prevention in patients with SCI and MS not merely as a clinical issue, but as a missed public health opportunity—one with implications for systems planning, health equity, and antimicrobial stewardship. The burden of UTIs in neurological patients using catheters is frequently underestimated in the current debate, yet our findings confirm that infections are not only common but often recurrent, severe, and preventable. Hospitalizations affected a significant portion of IC users in some studies [11], likely reflecting not a failure of the technique itself, but rather the absence of standardized, condition-specific care pathways. In particular, dedicated guidelines for catheter management in patients with MS are scarce, and the lack of structured protocols may contribute to under-recognition of the problem [10].

The review also brings attention to underexamined dimensions of care, including the psychosocial distress and dissatisfaction with urinary Quality of Life reported by many patients. When IC is poorly supported, it often leads to abandonment of the method in favor of less effective alternatives. This undermines the known benefits of IC and perpetuates the very complications it is designed to prevent. Critically, the near-absence of MS-specific studies exposes a significant evidence gap, raising concerns about the generalizability of SCI-based models to other neuro-urological conditions. These findings point to the need for tailored, equity-oriented interventions that recognize contextual factors and patient-specific challenges.

Given the international scope of the studies included, the issues identified are not isolated or system-specific. They underscore the need for coordinated action—through context-sensitive implementation strategies and a stronger alignment between clinical practice and real-world patient needs.

Future strategies must prioritize comprehensive patient education and regular follow-up—specifically annual bladder function examinations—to detect high intravesical pressures early and adjust catheterization frequencies before complications arise.

### 4.4. Strengths and Limitations of This Study

Through a comprehensive search strategy, this review offers valuable insights into the epidemiological burden of UTIs among adult patients with SCI and MS using IC at the international level. Understanding the impact of UTIs in these populations is critical for guiding effective preventive strategies and improving clinical management. In particular, the identification of major risk factors would enhance our understanding of the condition and represent a fundamental step toward the development of targeted interventions. Importantly, the findings of this review may inform evidence-based public health planning and support strategic decision-making to safeguard the health and well-being of these vulnerable groups. Moreover, this study underscores the need for further investigation, particularly regarding the standardized definition of UTIs, the role of specific catheter types, and the identification of risk factors unique to each target population.

Several limitations should be considered when interpreting the findings of this review. First, no formal quality assessment was conducted, and the lack of stratified data in many studies restricted the depth of subgroup analyses; additionally, the predominance of observational designs limited the ability to infer causality and suggests that the reported estimates should be interpreted with caution. Second, the inclusion was restricted to articles published in English. Although the screening process was conducted in accordance with the PRISMA guidelines, the potential for selection bias cannot be entirely ruled out [54]. Furthermore, despite implementing a thorough search strategy, substantial heterogeneity among the included studies—in terms of study design, study populations, outcome reporting, follow-up duration, and the criteria used to define UTIs—emerged. Our review confirms substantial heterogeneity in UTI definitions among IC users with SCI and MS. Only a minority [23,24,25,26,32,34] of included studies explicitly required both symptoms and microbiological confirmation, while most used broader or self-reported outcomes, which may conflate asymptomatic bacteriuria with symptomatic infection and inflate UTI rates. Furthermore, the thresholds used to define recurrent UTI (e.g., ≥3 episodes/year vs. alternative cutoffs) differ across studies [18,38], mirroring the variability described in prior neuro-urology literature and further limiting cross study comparability.

The evidence base remains heavily skewed toward SCI populations. Only one study focused explicitly on MS, raising concerns about the representativeness of current models and reinforcing the need for more inclusive, condition-specific research. Despite these limitations, the review identifies clear and recurring patterns—especially in infection frequency, hospitalization rates, and the structural barriers undermining IC implementation.

Future studies should address these gaps by adopting standardized UTI definitions [52] (e.g., EMA/FDA criteria [55,56]), applying longitudinal designs, and incorporating patient-reported outcomes and lived experience. Crucially, research must move beyond procedural comparisons to evaluate system-level interventions and contextual determinants that shape IC outcomes in practice.

## 5. Conclusions

This scoping review highlights the persistent and often underestimated burden of UTIs in individuals with NLUTD who rely on IC, particularly those with SCI and MS. While the available literature supports IC as a clinically effective and patient-preferred method for bladder management, its real-world implementation remains fraught with barriers that undermine its potential benefits. High rates of UTIs, recurrent infections, and related hospitalizations persist not because of the technique itself, but because of systemic shortcomings in access, hygiene education, caregiver support, and continuity of care.

The evidence reviewed confirms that IC outcomes are deeply dependent on the context in which they are delivered. Access to appropriate catheter types—especially single-use or hydrophilic models—along with structured caregiver and patient education, ongoing follow-up, and attention to the psychological and social dimensions of catheter use are all critical to reducing infection rates. Yet these elements are often inconsistently provided or entirely absent, particularly in low-resource settings.

Moreover, the current evidence base is overwhelmingly centered on SCI populations, with a striking lack of data on patients with MS. This imbalance raises concerns about the generalizability of existing findings as assumptions drawn from SCI-focused research may not apply equally across different neurological conditions. This underrepresentation contributes to the underutilization of IC in certain groups and limits the development of targeted, patient-centered strategies.

Future research should address these gaps by employing standardized UTI definitions, consistent outcome reporting, and inclusive study designs that reflect the full diversity of affected populations. Crucially, the effectiveness of IC cannot be separated from the system that supports it. Public health policy must move beyond the technical endorsement of IC and invest in fully integrated, person-centered programs that include education, access to appropriate resources, and follow-up. Such approaches would not only reduce the incidence of preventable complications like UTIs, but also enhance long-term health system sustainability, support responsible antibiotic stewardship, and promote equity in the delivery of neurological care.

In conclusion, UTIs in IC users with neurological disorders represent not just a clinical challenge but also a broader public health concern. Addressing this silent burden requires coordinated action at clinical, systemic, and policy levels to ensure that the proven benefits of intermittent catheterization translate into meaningful outcomes for all patients.

## Figures and Tables

**Table 1:** PICO framework.

P (population):	Adults (≥18 years) with NLUTD due to SCI or MS who use IC.
I (intervention):	Intermittent catheterization (particularly clean intermittent catheterization, CIC), and exposure to various user-dependent, technique-related, and catheter-specific factors.
C (comparator):	The comparison varies by study and includes the absence of a specific risk factor or the use of an alternative catheterization practice (e.g., single-use vs. reusable, self-IC vs. assisted-IC, and educated caregiver vs. standard care).
O (outcomes):	Primary: Epidemiological measures of UTI burden (e.g., incidence, prevalence, frequency per year, recurrence rates). Secondary: Associated healthcare utilization (e.g., hospitalizations) and identified risk factors for UTIs.

**Table 2:** General characteristics of the primary studies included in the review.

First Author. Year and Country	Study Design	Underlying Pathology	Target Population	IC Type/Managment	IC Frequency	IC Related Burden of Disease
Chen S.F., 2014 Taiwan [15]	Cross-sectional survey	SCI	Clean Intermittent Catheterization (CIC) group: 163 patients (18.2)	CIC/Self-CIC	N.A.	Prevalence of (Patient Reported Urinary Tract Infections) PRUTIs during the previous 3 years: 31 patients (41.3% of the CIC group that underwent urinalysis)
Yıldız N., 2014 Turkey [14]	Cross-sectional observational study	SCI	IC group: 243 M: 178 (73.2%) F: 65 (26.8%)	Aseptic IC	N.A.	Prevalence of UTIs related to aseptic IC: 51 patients (21.9% of the observed aseptic IC users)
Yilmaz B., 2014 Turkey [3]	Retrospective observational study	Acute SCI: 88 patients Chronic SCI: 119 patients	CIC group: 207	CIC	N.A.	UTIs prevalence: Symptomatic UTIs: 76/207 patients (36.7%)
Krassioukov A., 2015 Canada [39]	Cross-sectional observational study	SCI	TOT: 61 patients Mean Age (SD): 35 ± 7.7 M: 53 (87%) F: 8 (13%)	CIC	Mean number of ICs: 6 ± 2 times per day (ranging from 1 to 10 per day). Re-use (19 patients). Mean number of IC using: 34 ± 50 times	UTI frequency: Single use IC: 1 ± 1 UTI/year Re-use IC: 4 ± 3 UTI/year UTIs in developing countries: 3.5 ± 2.8/year UTIs in developed countries: 1.6 ± 2/year
Rabadi M.H., 2015 USA [30]	Retrospective observational study	SCI with NB	CIC group: 40 patients	CIC	N.A.	UTI prevalence: 14 (35.0%)
Mukai S., 2016 Japan [28]	Retrospective observational study	SCI	TOT: 259 patients Median age: 47 (range 12–90 years) M: 220 (84.9%) F: 39 (15.1%)	CIC	Median number of CIC per day: 7 (Range: 1–20)	UTI prevalence: 129 (49.8%) Febrile UTIs during follow-up period: 67 (25.8%)
Alavinia S.M., 2017 Canada [20]	Prospective observational study	Subacute SCI	CIC group 40 (72.70%)	Self CIC: 25 Assisted CIC: 15	N.A.	UTI incidence in CIC: 26 (65.0%)
Stillman M.D., 2018 USA [13]	Secondary analysis of data from a prospective clinical trial	Traumatic SCI	IC group At baseline: 35 3 months: 31 6 months: 35 9 months: 38 12 months: 39	IC	N.A.	UTI incidence rate: At baseline: IC: 13/35 (37%) 3 months: IC: 19/31 (61%) 6 months: IC: 16/35 (46%) 9 months: IC: 13/38 (34%) 12 months: IC: 12/39 (31%)
Crescenze I., 2019 USA [22]	Prospective cohort study	SCI	CIC: 753 Non-self: 82 (10.9%) M: 67.1% F: 32.9% Mean age 43.2 (18–86)	Self CIC:671 Assisted CIC: 82 (10.9%)	Mean duration of CIC 9.5 years (0–44)	PRUTI reported per year >4: 209 patients (27.8%)
McClurg D., 2019 UK [10]	Three-part mixed-method study: Prospective observational study, qualitative interviews, retrospective survey.	MS	TOT CIC: 56 Continuers group (at 1 year follow up): 43 patients M: 12 (28%) F: 31 (72%) Mean Age (SD): 49.9 (12.5) Discontinuers: 13 patients M: 2 (15%) F: 11 (85%) Mean Age (SD): 51.3 (10.1)	Self-CIC	N.A.	Prevalence At baseline: Discontinuers (*n* = 13) reported UTIs: 3 (23%) Continuers (*n* = 43) reported UTIs: 22 (51%) At 8 months: Discontinuers: No UTI: 6 (46%) Same number of UTIs: 0 (0%) Increased UTIs: 7 (54%) Decreased UTIs: 0 (0%) Continuers: No UTIs: 6 (46%) Same number of UTIs: 11 (26%) Increased UTIs: 7 (54%) Decreased UTIs reported: 3 (7%)
Huang X. 2019 China [33]	Randomized clinical trial	SCI with NB	TOT: 80 patients M: 49 F: 31 Quality Control Circle group: 40 patients M: 25 F: 15 Mean Age (SD): 56.7 ± 4.3 years CG: 40 patients M: 24 F: 16 Mean (SD): 57.3 ± 4.8 Age years	Self-CIC	N.A.	UTI incidence Quality Control Circle Group: 4 patients (10%) Control Group: 13 patients (32.5%)
Roth J.D. 2019 USA [21]	Retrospective survey	SCI	CIC group: 753 Mean age (SD): 43.7 (13.1) years M: 504 (66.9%)	CIC	Mean number of daily catheterizations: 5.94 (SD 1.81).	PRUTI during the previous year: 0: 172 (22.8%) 1–3: 372 (49.4%) 4–6: 117 (15.5%) 6: 92 (12.2%) Adjusted odds of increased UTI frequency 3.42 (2.25–5.18) for CIC (reference spontaneous voiding)
Anderson C.E., 2019 Switzerland [23]	Prospective cohort study	SCI	IC group: 73 (19.8%)	Assisted-IC: 41 patients (11.1%) Self-IC: 32 patients (8.7%)	N.A.	UTI prevalence 28 days after admission Assisted IC Patients with 1 UTI: 12/41 patients (29.3%) Patients with ≥2 UTIs: 13/41 (31.7%) Self-IC Patients with 1 UTI: 8/32 (25.0%) Patients with ≥2 UTIs: 6/32 (18.8%) UTIs IR and IRR (reference Spontaneous Voiding): IC-assisted: Crude IR per 100 person-days (95% CI): 0.68 (0.52–0.90) Unadjusted IRR (95% CI): 6.16 (3.04–12.50) Adjusted IRR (95% CI): 6.05 (2.63–13.94) IC-self: Crude IR per 100 person-days (95% CI): 0.53 (0.39–0.71) Unadjusted IRR (95% CI): 4.50 (2.25–9.01) Adjusted IRR (95% CI): 5.16 (2.31–11.52)
Hennessey D., 2019 Australia [24]	Prospective observational study	SCI	ISC group: 45	ISC	N.A.	UTIs/1000 days: 6.84
Farrelly E., 2020 Sweden [26]	Prospective cohort study	Post-traumatic SCI	CIC group: 157 (38%)	CIC	N.A.	Mean number of UTIs during the previous year: 2.5 UTIs/year: 0: 58 (36.9%) 1–3: 57 (36.3%) 4–6: 26 (16.5%) >6: 16 (10.2%)
Nade E.S. 2020 Tanzania [16]	Cross-sectional pilot study	SCI	CIC patients: 23	Self -IC: 8 (16%). Family member assisted IC: 15 (31.3%)	N.A.	Incidence of Febrile UTIs: 9 (39.2% of all patients performing CIC) Inpatients: 4 (25%) Outpatients: 5 (71%).
Patel D.P., 2020 USA [11]	Cross-sectional observational study nested within a registry-based observational study	SCI	TOT: 176 patients M: 110 (63%) F: 66 (37%) Mean age (SD): 45.3 (12.8) years	CIC	N.A.	PRUTI frequency during previous year (in 176 patients that discontinued CIC): 0: 17 (26%) 1–3: 79 patients (45%) ≥4: 60 patients (34%) Hospitalization for UTI during the previous year: 29 (16%)
Neyaz O., 2020 India [25]	Prospective observational study	SCI	TOT: 31 patients M: 29 (93%) F: 2 (7%) Mean Age (SD): 28.6 ± 9.2 years	Self-CIC	N.A.	Incidence of Symptomatic UTIs: 2.29 episodes per patient per year
Theisen K.M., 2020 USA [27]	Prospective observational study	SCI	CIC group: 780	CIC	N.A.	PRUTI frequency during previous year: 0: 178 (46%) 1–3: 381 (49%) 4–6: 127 (16.7%) 6: 94 (12.4%)
Kim Y., 2021 South Korea [32]	Retrospective descriptive study	SCI	TOT: 964 M: 742 (77.0%) F: 222 (23.0%) Mean Age 46.1 (15.6)	Self CIC: 54 (5.6%) Caregiver assisted CIC: 64 (6.6%)	N.A.	UTI prevalence in Self CIC group: 12 (22.2%) UTI prevalence Caregiver assisted CIC: 13 (20.3%)
Walter M., 2021 Canada [17]	Cross sectional survey	SCI	IC group: 109 (84%)	IC	Median duration of IC: 10 years (IQR 6–15, range 1–28). Median frequency of catheterizations per day: 5 (IQR 4.5–6, range 1–10).	PRUTI incidence during previous year: 69 (63% of IC patients) Median number of PRUTIs per year: 1 (IQR 0–2, range 0–12).
Berger A., 2022 Multicentric (Germany, Netherlands) [31]	Retrospective Chart Review	SCI	IC patients 23 (31.5%)	Caregiver assisted IC	N.A.	UTI incidence at 3 months follow-up: 42 patients (57.5%) UTI incidence per 100 person-month: 31.46
Chen S.F., 2022 Taiwan [38]	Retrospective observational study	SCI	CIC group: 81 patients	CIC/Self-CIC	N.A.	Recurrent UTI incidence: 57 (70.4%)
Xing H., 2023 China [29]	Retrospective Chart review	SCI	TOT 183 patients M: 123 (67.2%) F: 60 (32.8%) Median Age: 49.0 Years (37–59 range)	CIC: 56 (30.6%) Self-CIC: 27 (69.4%)	IC performed 4–6 times/day with disposable catheters	UTI incidence: 44 (24.0%) Occurrence rate of UTI: 1.31 (95% Cis, 0.96–1.77) events per 100 person-days
Liu J., 2023 China [18]	Cross-sectional observational study	SCI	CIC group: 623	CIC	Frequency of CIC use (times per day): <1: 116 (18.6%) 1–6: 478 (76.7%) >6: 29 (4.7%)	Incidence UTIs: 525 (84.3%) Recurrent UTIs: 333 (53.5%) Number of outpatient visits for UTIs:3 (0.4% of total CIC users) Number of hospitalizations for UTIs:12 (1.9%) CIC use < 1/day (*N* = 116): UTIs: 107 (20.4%) OR (reference 1–6): 1.333 (95% CI: 0.595–2.988, *p* = 0.485) CIC use 1–6/day (*N* = 478): UTIs: 391 (74.5%) OR: 1 (Reference) CIC use > 6/day (*N* = 29): UTIs: 27 (93.1%) OR (reference 1–6): 2.378 (95% CI: 0.532–10.626, *p* = 0.257)
Sekido N. 2023 Japan [19]	Cross Sectional Internet Survey	SCI	ISC group: 247 M: 185 (74.9%) F: 62 (25.1%) Mean Age (SD): 47.8 ± 14.9 years	Reusable silicone catheter: 136 (55.1) Single-use catheter: 111 (44.9)	N.A.	UTI incidence ISC (247) Symptomatic UTI 129 (52.2%) Febrile UTI 82 (33.2%) Hcp-sUTI 96 (38.9%) Re-usable (136) Symptomatic UTI 75 (55.1%) Febrile UTI 46 (33.8%) Hcp-sUTI 58 (42.6%) Single Use (111) Symptomatic UTI 54 (48.6%) Febrile UTI 36 (32.4%) Hcp-sUTI 18 (34.2%) UTI episodes ISC (SD) Symptomatic UTI 2.8 (4.95) Febrile UTI 0.9 (2.14) Hcp-sUTI 1.5 (2.83) Re-usable (SD) Symptomatic UTI 2.8 (4.18) Febrile UTI 0.8 (1.43) Hcp-sUTI 1.5 (2.45) Single use (SD) Symptomatic UTI 2.8 (5.77) Febrile UTI 1.0 (2.77) Hcp-sUTI 1.4 (3.26)
Milicevic s., 2024; Serbia [34]	Retrospective study	SCI	TOT: 369	Self IC: 287 Assisted IC: 72	N.A.	UTI incidence: 252 (62.7%) Self IC 69 (17.2%) Assisted IC
Ali S., 2024; Saudi Arabia [35]	Retrospective cohort study	SCI	TOT: 1000 Gender: M: 87.2% F:12.8% F Mean age: 34.07y (13.19)	524 (52.4%) hydrophilic coated catheters (HCC), 476 (47.6%) PVC uncoated catheters (PUC)	N.A.	Symptomatic UTI incidence HCC 46.60% PUC 79.40%
Luo J., 2024; China [36]	Retrospective Case Control study	SCI	TOT: 84 Control group 40 Observation group 44	Self-CIC	N.A.	UTI prevalence: Control Group: 20 (50%) Observation Group: 11 (25%)
Cheng T.C., 2024; Taiwan [37]	Retrospective cohort study	SCI	TOT: 47 M 38 (80.9%) F 9 (19.1%) Mean age: 47y	CIC	N.A.	UTI incidence per month: 0.03 UTIs/month

CIC Clean Intermittent Catheterization, F Female, Hcp-sUTI Healthcare provider-diagnosed symptomatic urinary tract infection, IC Intermittent Catheterization, IQR Interquartile Range, IR Incidence Rate, IRR Incidence Rate Ratio, ISC Intermittent Self Catheterization, M Male, NB Neurogenic Bladder, OR Odds Ratio, PRUTI Patient Reported Urinary Tract Infections, SCI Spinal Cord Injury, SD Standard Deviation, UTI Urinary Tract Infections; N.A.: not available.

**Table 3:** Urinary Tract Infections Risk Factors mentioned in the studies categorized by Kennelly’s Urinary Tract Infections Risk Factor Model [8].

Study	General Conditions	Intermittent Catheterization	User Compliance/Adherence	Local Urinary Tract Conditions
Krassioukov A., 2015 [39]	-	-	Procedure-related (catheter reuse)	-
Mukai S., 2016 [28]	Gender (male); neurological status (ASIA scale C or worse)	-	-	-
McClurg D., 2019 [10]	-	-	CIC Compliance (continuation use, discontinuation use)	-
Anderson C.E., 2019 [23]	-	-	Hygienic Procedure (intermittent self catheterization, assisted catheterization)	-
Kim Y., 2021 [32]	-	Catheter type (indwelling, suprapubic catheters)	-	-
Liu J., 2023 [18]	Gender (male); disease duration; urinary incontinence	Change in catheterization method	Difficulty with insertion; infrequent CIC; catheter reuse (borderline)	-
Sekido N., 2023 [19]		Catheter type (indwelling catheters)	Procedure-related (catheter reuse)	-
Milicevic S., 2024 [30]	Impaired Bladder Compliance (reflex voiding, and spontaneous voiding)	Catheter type (indwelling catheters)	Hygienic Procedure (intermittent self catheterization, assisted catheterization)	-
Ali S., 2024 [35]	-	Catheter type (hydrophilic-coated catheters, PVC catheters)	-	-
Luo J., 2024 [36]	-	-	Lack of caregiver education and support	-

## Data Availability

The original contributions presented in this study are included in the article. Further inquiries can be directed to the corresponding authors.

## Supplementary Materials

The following supporting information can be downloaded at: https://www.mdpi.com/article/10.3390/diseases14020058/s1, Table S1: PRISMA 2020 Checklist.
